# Multi-Scale Modelling of Aggregation of TiO_2_ Nanoparticle Suspensions in Water

**DOI:** 10.3390/nano12020217

**Published:** 2022-01-10

**Authors:** Giulia Mancardi, Matteo Alberghini, Neus Aguilera-Porta, Monica Calatayud, Pietro Asinari, Eliodoro Chiavazzo

**Affiliations:** 1 Energy Department, Politecnico di Torino, Corso Duca degli Abruzzi 24, 10129 Torino, Italy; matteo.alberghini@polito.it (M.A.); pietro.asinari@polito.it (P.A.); eliodoro.chiavazzo@polito.it (E.C.); 2 Clean Water Center, Corso Duca degli Abruzzi 24, 10129 Torino, Italy; 3 Laboratoire de Chimie Theorique, CNRS, Sorbonne Université, 4 Place Jussieu, 75005 Paris, France; neusaguilera@gmail.com (N.A.-P.); calatayu@lct.jussieu.fr (M.C.); 4 Istituto Nazionale di Ricerca Metrologica, Strada delle Cacce 91, 10135 Torino, Italy

**Keywords:** Density Functional Theory, Molecular Dynamics, Umbrella Sampling, Brownian dynamics, multiscale, nanoparticle, aggregation, clustering

## Abstract

Titanium dioxide nanoparticles have risen concerns about their possible toxicity and the European Food Safety Authority recently banned the use of TiO_2_ nano-additive in food products. Following the intent of relating nanomaterials atomic structure with their toxicity without having to conduct large-scale experiments on living organisms, we investigate the aggregation of titanium dioxide nanoparticles using a multi-scale technique: starting from ab initio Density Functional Theory to get an accurate determination of the energetics and electronic structure, we switch to classical Molecular Dynamics simulations to calculate the Potential of Mean Force for the connection of two identical nanoparticles in water; the fitting of the latter by a set of mathematical equations is the key for the upscale. Lastly, we perform Brownian Dynamics simulations where each nanoparticle is a spherical bead. This coarsening strategy allows studying the aggregation of a few thousand nanoparticles. Applying this novel procedure, we find three new molecular descriptors, namely, the aggregation free energy and two numerical parameters used to correct the observed deviation from the aggregation kinetics described by the Smoluchowski theory. Ultimately, molecular descriptors can be fed into QSAR models to predict the toxicity of a material knowing its physicochemical properties, enabling safe design strategies.

## 1. Introduction

Materials with characteristic size in the range of 1–100 nm are commonly defined as nanomaterials and play a crucial role in a number of fields ranging from the energy sector [1,2,3] up to drug delivery and biomedicine [4,5]. As such, in the last years, the availablity of nanosized materials in commercial products (e.g., sunscreen and food additives) has raised the concern about their toxicological effects [6]. Despite a large number of experiments, it is difficult to establish clear trends in structure-toxicological endpoints because of the multiple factors regulating cell uptake and toxicity of nanoparticles. Dimension, degree of crystallinity, shape, aspect ratio, and ability to aggregate, may result in different levels of toxicity. To address this problem, several research projects recently funded by the European Union are currently ongoing, among which stand out for relevance NanoInformaTIX [7] and NanoSolveIT [8]. The project partners of both projects work towards the organization of the huge amount of values of toxicological endpoints already available and carry on other experiments with a clear rationale to fill the gaps in the literature, with the final scope to create a platform for easy consultation of the data. Nanomaterials raising health and safety concerns can be grouped into four broad categories: oxides such as titanium and silicon dioxide, soluble materials such as zinc oxide, metals such as gold and silver, and carbon-based nanomaterials such as carbon fibers and nanotubes. The present work is part of the NanoInformaTIX project and is focused on calculating physicochemical descriptors, in this case, nanoparticle free energy of aggregation and clustering, to be fed into Quantitative Structure-Activity Relationship models (QSAR) to finally predict toxicity. QSAR models are based on correlation existing between molecular properties (descriptors) of substances and their toxic endpoints, which allows predicting unknown toxic endpoints for substances known their molecular descriptors [9]. One of the main tasks of the partners involved in NanoInformaTIX is to find suitable descriptors for QSAR models providing atomic/molecular information useful for toxicity prediction. Recently used descriptors for oxide nanoparticles involve energetic, geometric, and electronic structure parameters such as total energy, bandgap, or composition of core-shell regions, and are obtained by ab initio and force-field classical modelling [10,11,12]. In this work, we provide descriptors obtained by different scales of calculations, from ab initio to Brownian dynamics, for a set of titania nanoparticles. In particular, we believe the *aggregation free energy*, calculated in this work using classical Molecular Dynamics (MD) simulations, provides valuable information on the behavior of such particles in water: it determines the speed of the aggregation and, therefore, the final size of the nanomaterial can reach, ultimately regulating cellular uptake [13,14]. Although computationally expensive, it could potentially be used as a descriptor in the QSAR models, in addition to the common ones. Moreover, for titanium dioxide nanoparticles with radius in the range 0.78–2 nm, there is a linear correlation between aggregation free energy and volume, additional free energy calculations will prove if this correlation exists also for larger nanoparticles and materials other than titanium dioxide.

In this work, we focus on titanium dioxide nanoparticles (NPs), whose possible toxicity after human consumption has recently raised concerns; for this reason, the simulations are carried out in the water, which is the main solvent in the body compartments. Titanium dioxide is widely used for its bright white color, stemming from the large bandgap of 3.2 eV [15], which makes the material absorb light in the UV region of the electromagnetic spectrum. Despite the large bandgap, which restricts the light absorption to the UV range, titanium dioxide is commonly employed as a photocatalyst for decomposition of organic pollutants, because it is chemically stable, low cost, and both its most common polymorphs anatase and rutile show an excellent photocatalytic activity [16]. It is also added to polymers to make them more resistant to UV light, reducing their fading and cracking. In automotive manufacturing, titanium dioxide is used as a protective coating for polycarbonates, which substitute metal and glass parts in lightweight vehicles [17]. Titanium dioxide is used as a white pigment in paints, papers, textiles, cosmetics, and foods; the European Food Safety Authority (EFSA), however, recently published an updated safety report for titanium dioxide, labeled as an E171 food additive, which states that titanium dioxide is not allowed anymore for human consumption due to possible genotoxic effects after ingestion [18]. E171 has ≈50% of particles with a diameter smaller than 100 nm, less than 1% of particles are below 30 nm [18,19]. Nano-TiO_2_ used in sunscreen is made of even smaller particles with a diameter in the range of 1 to 150 nm; to avoid cellular damage from the reactive oxygen species produced during titanium dioxide photocatalytic activity, nanoparticles are commonly coated with alumina or silica, which also improve dispersion [20]. The small size of nanomaterials increases enormously the surface-to-bulk ratio, changing dramatically the macroscopic properties of the material. Due to their size, nanomaterials are hard to characterize experimentally, whereas simulations can be of great help to shed light on the physicochemical properties. However, such nanoparticles are too large to be simulated at a quantum level, hence it is necessary to make some approximations, the most common one is to consider a planar slab representative of the material [21,22]. The main problem of this approximation is that nanospheres, nano ellipsoid, and nanorods have a broader range of uncoordinated Ti sites than nanocrystals and slabs [23], furthermore, nanoparticles present a wide range of low index facets because of their curvature, so considering a single, low index slab can lead to different results, especially in an aqueous environment, where water molecule can absorb on the surface in a molecular, dissociative or even a mixture of the two ways [24,25].

The present work develops a multiscale modeling approach aiming to predict the aggregation kinetics of colloidal suspensions of nanoparticles based on their atomistic description. The proposed method synergistically combines classical MD and Brownian dynamics (BD) simulations: the former was used to obtain the pairwise interaction potential (PMF) between identical NPs, which is then used as input in the latter to simulate the aggregation of a large set of NPs. Ab initio Density Functional Theory (DFT) is used in selected structures to characterize the energetics of bare and solvated particles; potential energies can be used as nanoparticle descriptors [12,26] First, the simulation protocol and the studied setups are described in the Section 2 in order to ensure the reproducibility of the obtained results. In the Section 3, the PMFs obtained via MD simulations, their fitting via polynomial functions, and the main results of BD simulations are reported. Finally, in the Section 4, the simulation results are compared with classical theoretical models of interaction and aggregation between NPs: the obtained PMFs are compared with DLVO theory, while the clustering process is compared with Smoluchowski aggregation theory. The obtained results show a deviation from the theoretical predictions, particularly when considering large volume fractions of NPs, thus the proposed modeling approach is essential to predict realistic behavior of dense suspensions of NPs.

## 2. Materials and Methods

### 2.1. Molecular Models and Strategy

**Figure 1:**
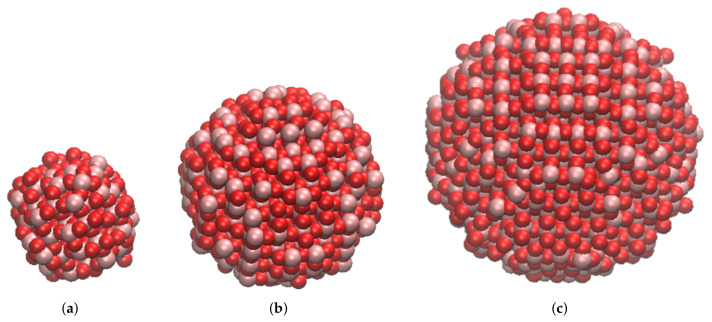
TiO_2_ nanoparticles used in this work: (**a**) Ti_111_O_222_, radius 0.78 nm; (**b**) Ti_417_O_834_, radius 1.5 nm; (**c**) Ti_985_O_1970_, radius 2 nm. Image obtained with VMD software [28], version 1.9.3.

We selected three spherical stoichiometric nanoparticles cut out of the bulk anatase crystalline structure with the web-based tool Vi-seem [27]: Ti_111_O_222_ of radius 0.78 nm, Ti_417_O_834_ of radius 1.50 nm and Ti_985_O_1970_ of radius 2.00 nm. They were chosen to be representative of small nanoparticles found in several commercial applications, and to be calculable by classical force fields MD. Figure 1 displays the models used.

The strategy of the present work is to characterize the behavior of the three NP configurations at different computational levels: ab initio DFT, classical MD, and BD. The structures are first equilibrated in a water box by means of classical MD, then the equilibrated structures are evaluated using ab initio DFT to assess energetics in a vacuum and in the presence of implicit water solvent, and, finally, BD simulations give insights into the aggregation process.

### 2.2. Ab Initio DFT Calculations

The VASP code version 5.4.4 was used to perform the DFT calculations [29]. Core electrons are represented by the projector-augmented wave method PAW [30] pseudopotentials H, Ti, and O${}_{s}$ (1, 4, and 6 valence electrons respectively), and a cutoff energy of 282 eV was used for the plane waves. The generalized gradient approximation (GGA) approach was applied for the exchange and correlation potential with the Perdew–Burke–Erzenhof (PBE) functional [31], spin non-polarized. The tests performed on the bulk anatase i.e., bulk geometry and thermodynamics, are in good agreement with the experimental values, see Table S3 in the Supporting Information. The energy convergence criterium was set to 0.1 meV for the electronic loop. The gamma-only version was used; a single point calculation on a snapshot obtained from the MD trajectory was computed. The nanoparticle models were put in a box with a minimum of 10 Å between images. The solvation energy in water was only computed for the smallest particles due to resources limitations, following the procedure described in [32].

### 2.3. Classical Molecular Dynamics Simulations

Classical MD simulations were performed using version 4.09 of the DL_POLY computational package [33] patched with version 2.5.1 of Plumed [34,35,36]. We started by placing a single nanoparticle in the center of a cubic box large enough to avoid interaction with the periodic images, the size of the cubic boxes were 5 nm to contain the 0.78 nm radius nanoparticle, 6 nm to contain the 1.50 nm radius nanoparticle, and 7 nm to contain the 2.00 nm radius nanoparticle. The box was filled with water molecules and duplicated along the *x* axis to get an orthorhombic box containing two identical TiO${}_{2}$ nanoparticles. We performed classical MD simulations in water because it is the solvent in biological environments; it is a widely used approximation [37,38,39] to body fluids, that are rich in ions, proteins, and lipids which are large molecules that would make MD simulations extremely expensive; moreover, their structure is not relevant in the present work. The simulation temperature was set at 310 K (body temperature) and the pressure at 1 bar. The timestep was set to 0.1 fs, the system cutoff was 8 Å, the Nosé-Hoover algorithm [40,41], with a relaxation time of 0.1 ps, was employed in both NVT and NPT ensembles to ensure consistency between the equilibration and the production runs. We used the force field of Matsui and Akaogi [42] for titanium dioxide, with the modifications introduced by Alimohammadi and Fichthorn for titania-SPC/E water interactions [43], as previously used by the authors [44]. Our MD protocol consisted of an initial 0.25 ns equilibration in an NVT ensemble (constant number of particles, constant volume, and constant temperature), followed by a second 0.25 ns equilibration in an NPT ensemble (constant number of particles, constant pressure, and constant temperature); the NPs were kept frozen during this equilibration, to adjust the water density at the experimental conditions. Afterward, the Umbrella Sampling (US) technique [45] was used to obtain the free energy profile (or Potential of mean Force, PMF) for the aggregation of the two NPs. This method makes use of a harmonic potential as a driving force to move NPs at a specific target distance, the PMF for the approaching can be calculated through the Weighted Histogram Analysis Method (WHAM) [46,47,48]. The US simulations were run in an NPT ensemble, to obtain the Gibbs free energy considering the simulation parameters is reported in Supplementary Table S1. The computational effort required by this technique is massive: a single US window runs 24 h on 32 cores of HPC@POLITO facility, a complete PMF, in this case, requires 13 simulations to be combined using the WHAM analysis.

The computed PMF accounts for the steric repulsion at a short distance and the attraction at longer distances. The depth of the potential well gives the aggregation free energy (AFE), located at $d_{AFE}$, and allows building the inter-particle potential. The free energy profiles for the aggregation of two identical NPs in water, calculated atomistically with the force field of Matsui and Akaogi [42], were fitted considering both the repulsive short-range part of the energy profile and the attractive long-range contribution. Both the attractive and repulsive contributions were modeled as 5-th order polynomials. Beyond the cutoff radius $r_{c}$, namely the maximum distance simulated in MD, the particle interaction was fitted by a logarithm function which asymptotically decays towards zero, namely predicting no interaction between the NPs. Thus, the fitting equation for each NP size can be described as:

(1) $$PMF=\left\{\begin{array}{cc} \sum_{i=0}^{5}\;a_{i}\;d^{i} & if\;d\leq d_{AFE}\;,\\ \sum_{j=0}^{5}\;b_{j}\;d^{j} & if\;d_{AFE}<d\leq r_{c}\;,\\ c_{1}\ln d+c_{0} & if\;d>r_{c}, \end{array}\right.$$

being *d* the center-to-center NPs distance.

### 2.4. Brownian Dynamics Simulations

The analytical curves fitting the PMFs were used to create three sets of table potentials, each with a discretization step of 2 × $10^{-3}$ nm, which were employed to perform BD simulations with GROMACS (version 2019.6) [49]. A similar approach has been successfully used by some of the authors to simulate the aggregation kinetic of alumina NPs [50]. Each NP is mapped to a single spherical bead and their mass was estimated from their stoichiometric definition, i.e., as the sum of the mass of the atoms forming the NP (see Figure 1), while their diameter is intrinsically expressed through the fitted PMF curve. The aggregation kinetic was investigated by solving Langevin’s equation, which adds friction and a noise term to the Newton’s equation [51]:

(2) $$\frac{d^{2}r_{i}}{dt^{2}}=-\gamma_{i}\frac{dr_{i}}{dt}+\frac{F_{i}\left(r_{i}\left(t\right)\right)}{m_{i}}+\omega_{i}\;,$$

where $m_{i}$ is the mass of the *i-th* nanoparticle, $r_{i}$ is its position at time *t*, $\gamma_{i}$ is a friction coefficient. $\omega_{i}$ is a random process with zero mean and no with past positions or velocity and its auto-correlation function is $\langle \omega_{i}\left(t\right),\omega_{j}\left(t+\tau \right)\rangle =2\gamma_{i}k_{B}Tm_{i}^{-1}\delta \left(\tau \right)\delta_{ij}$, Ref. [52] where $k_{B}$ is the Boltzmann constant, and *T* is the reference temperature of the system, δ(τ) is the delta function and $\delta_{ij}$ is the Kronecker delta function. The friction coefficient $\gamma_{i}$, expressed in ps${}^{-1}$, accounts for the particle-solvent interactions and was evaluated according to the Stoke’s relation as:

(3) $$\gamma_{i}=\frac{6\pi \mu R_{i}}{u}$$

where $R_{i}$ is the particle radius, μ is the dynamic viscosity of the fluid at the reference temperature *T* and the constant *u* = 1.66054 × $10^{-27}$ kg was used for the conversion to atomic mass units. In GROMACS, Equation (2) is implemented as a difference equation for the particle velocity $v_{i}$ over a small time-step Δt by applying the friction and noise terms as an impulse [53]. Thus, Equation (2) can be re-written in its differential form as [49]:

(4) $$\begin{array}{c} \begin{array}{cc} \; & v_{i}^{\prime }=v\left(t-\Delta t/2\right)+\frac{F_{i}\left(t\right)}{m_{i}}\Delta t\;,\\ \; & \Delta v_{i}=-\left(1-\exp\left(-\gamma_{i}\Delta t\right)\right)v_{i}^{\prime }+\omega_{i}^{G}\sqrt{\left(1-{\left(1-\exp\left(-\gamma_{i}\Delta t\right)\right)}^{2}\right)\frac{k_{B}T}{m_{i}}\Delta t}\;, \end{array} \end{array}$$

where $\omega_{i}^{G}$ is a zero-mean Gaussian distributed noise with unitary variance. Simulations were run for at least 30 μs, using periodic boundary conditions, with a timestep of 0.1 ps and a temperature of 310 K, to keep consistency through scales. We used a 100 nm × 100 nm × 100 nm cubic box, while the number of simulated particles, randomly distributed in the simulation domain, was changed to match the target volume fraction ϕ. Four different volume fractions were tested for each NP size: 0.8%, 1.8%, 3.5%, and 7.0%. A detailed list of the parameters used for each simulation is listed in Supplementary Tables S4 and S5. Each simulation ran on 64 cores of the HPC facility for 800 to about 2000 h (1 to 3 months), depending on the considered volume fraction.

The size and population of the clusters formed during the simulations were analyzed via an in-house MATLAB^®^ post-processing algorithm. The proposed implementation does not require the *a priori* knowledge of the number of clusters present in the simulation box, which are in fact determined by the pairwise distance between each particle. Two particles were considered as neighboring and belonging to the same aggregate if their distance is less than a threshold distance, here considered to be 3R. Thus, two neighboring NPs must be in the attractive region of their interaction potential, while a third particle cannot be interposed between them.

The distance between two particles Δ was evaluated using Euclidean distance:

(5) $$\Delta =\sqrt{dx_{ij}^{2}+dy_{ij}^{2}+dz_{ij}^{2}}\;,$$

while periodic boundary conditions were considered when evaluating each component of the distance between two particles *i* and *j*, namely:

(6) $$dx_{ij}=x_{i}-x_{j}-h_{box}\left\lceil \frac{x_{i}-x_{j}}{h_{box}}\right\rfloor \;,$$

where the notation ⌈x⌋ stands for the round operator and $h_{box}=100$ nm is the size of the considered cubic box. Through Equations (5) and (6), also the clusters that straddle the boundary of the simulation box can be considered as included in the same aggregate.

## 3. Results

The section is organized as follows: first, we report the energetic properties of the nanoparticles obtained ab initio, then we report the aggregation free energy profiles obtained through Classical MD and US technique; last, we show the NP aggregation and clustering observed in the BD trajectories.

### 3.1. Ab Initio DFT Characterization

A snapshot was selected from the MD simulations to calculate ab initio properties of each nanoparticle model. Due to computational limitations, the energy of the particles was obtained as single-point in a vacuum, and in the presence of implicit water. Table 1 collects the different properties obtained in the single-point calculations. First, the ab initio energy was computed and used to evaluate the standard formation energy $\Delta H_{f}^{\circ }$, which is −8.28, −8.45 and −8.86 eV for the 0.78 nm, 1.50 nm, and 2.00 nm, respectively. Compared to the bulk value, −9.46 eV, there is a clear thermodynamic stabilization as the size increases, yet not reaching the bulk value. As for the solvation energies in water, the values of −0.42 eV and −0.34 eV are moderate (the value for a water molecule is −0.32 eV), indicating a better stabilization upon solvation for the smaller particle. Table 1 summarizes selected physico-chemical properties of the models together with ab initio energies.

### 3.2. Potential of Mean Force for the Approach of Two Identical Titanium Dioxide Anatase Nanoparticles in Water

**Figure 2:**
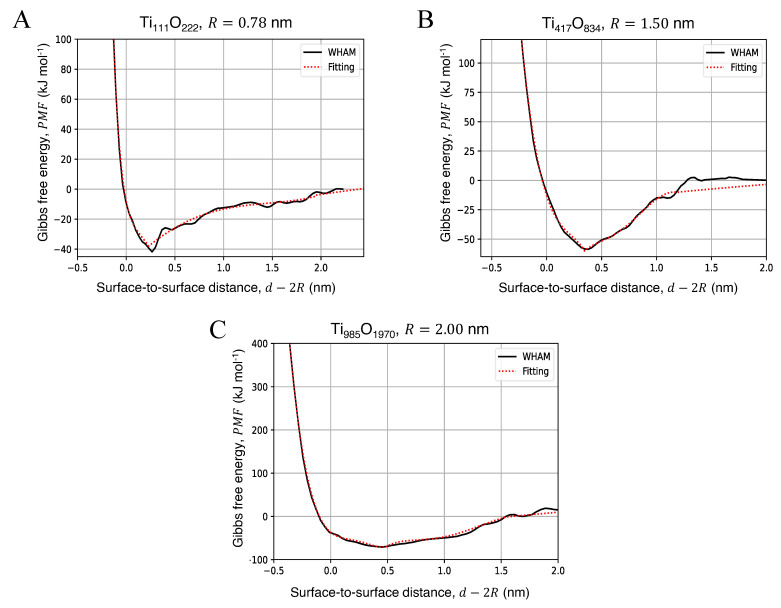
Free Energy profiles for the aggregation of two TiO_2_ NPs in water with chemical formula (**A**) Ti_111_O_222_, (**B**) Ti_417_O_834_ and (**C**) Ti_985_O_1970_. The results of the MD simulations (solid black line) were fitted with Equation (1) (dotted red line) to produce the pair-wise tabled potentials. The PMFs and their respective fit curves were represented as down-shifted considering a null interaction at d→∞.

The free energy profiles for the approaching of two identical NPs in water are presented in Figure 2, where the black curve is given directly using the WHAM analysis on the US trajectories. The red dashed line were fitted with Equation (1) by the least-squares methods and were used to perform the BD simulations (see Section 2.4). The fitting coefficients obtained for each configuration are reported in Supplementary Table S2. The corresponding aggregation free energies (AFE), calculated as the depth of the minimum of the potential well with respect to the energy at a non-interacting distance, and its distance with respect to the NP surface ($d_{AFE}-2R$) are reported in Table 2. Increasing the diameter of the NP, the potential well becomes wider, and the minimum of the free energy curve shifts towards a longer distance along the *x* axis, approximately 0.25 nm–0.5 nm beyond the NPs surface, which corresponds to the sum of the van der Waals radii of the surface atoms of the two NPs in contact. From Table 2 and Supplementary Figure S1, it is possible to see that the AFE increases with particle size; considering the particle volume, the size-free energy relationship is linear in the size range considered in this work.

### 3.3. Aggregation of Titanium Dioxide Anatase Nanoparticles Using Brownian Dynamics

**Figure 3:**
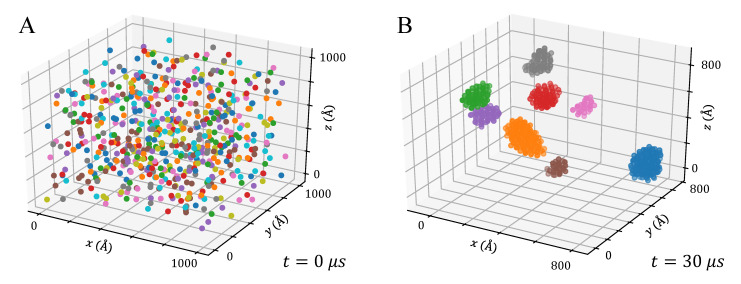
Formation of clusters during a BD production run of 600 NPs of Ti_417_O_834_ with ϕ = 0.8%, reported as an example; (**A**) Initial configuration with randomly distributed and isolated NPs; (**B**) After 3 μs, the particles formed 8 major aggregates. For a detailed view on xy, xz and yz planes, see Figure S2.

The fitted PMF curves were used to perform BD simulations and retrieve the aggregation kinetics of the simulated anatase NPs at four different volume fractions. All the configurations tested were initially composed of randomly distributed NPs (see Figure 3A, 600 NPs) and presented the progressive formation of aggregates, mostly spherical and ellipsoidal or, occasionally, slightly branched ellipsoids (see Figure 3B). These results are in agreement with previous experimental observations, where spherical TiO_2_ NPs in water were found to form branched aggregates [55].

In general, the more concentrated is the solution (namely, higher values of ϕ), the faster is the onset of the clustering process given the higher frequency of collisions.

**Figure 4:**
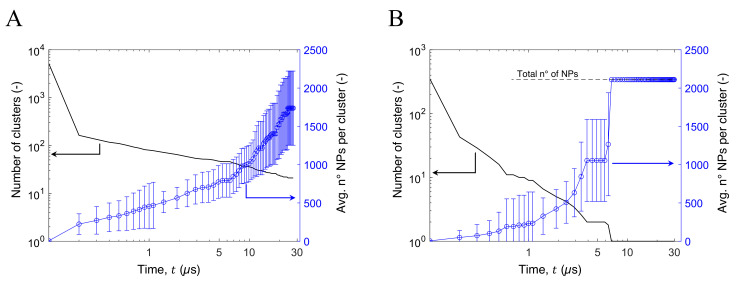
Aggregation kinetic of (**A**) Ti_111_O_222_, and (**B**) Ti_985_O_1970_ with ϕ = 7%, reporting the total number of clusters present in the simulation box (solid black line) and their average size (blue circles). Note that in the latter the NPs formed a single cluster after approximately 7 μs of simulations.

**Figure 5:**
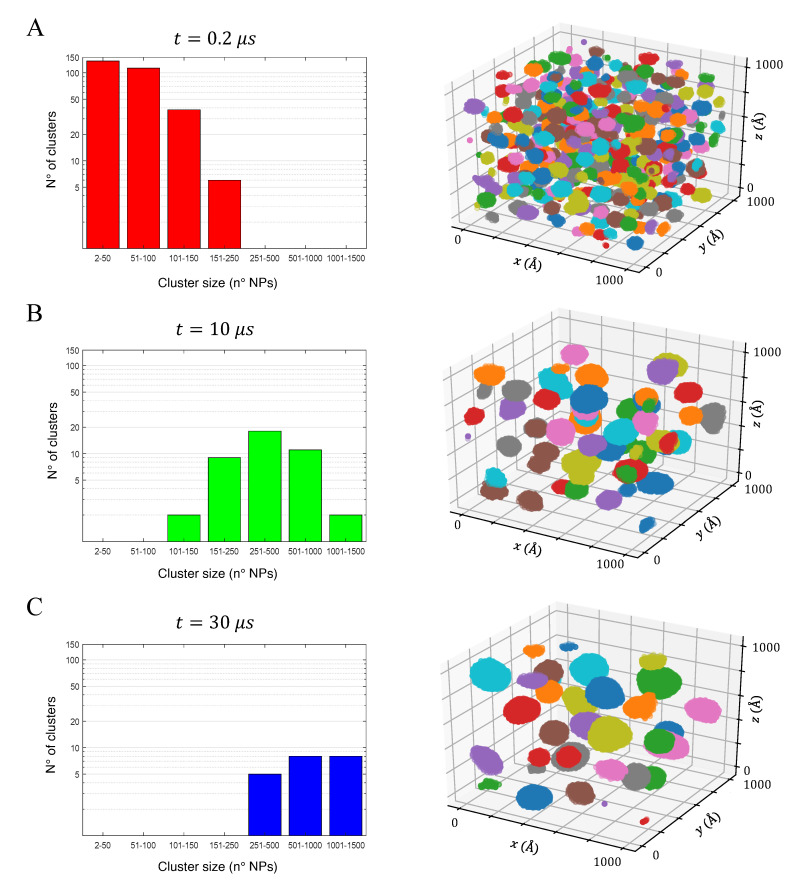
Aggregation kinetic of Ti_111_O_222_ with ϕ = 3.5% at (**A**) 0.2 μs, (**B**) 10 μs and (**C**) 30 μs. As the simulation advances, the clusters size distribution (histograms, left-hand side) tends towards the equilibrium configuration, namely fewer and larger clusters. Note that the aggregates were colored for simplicity of representation only.

The clustering process can occur through three different mechanisms: (i) collision of a single particle with another single-particle; (ii) collision of a single particle with a cluster; (iii) collision of two clusters. Figure 4 and Figure 5 show the aggregation kinetic of some of the tested configurations and can be used to highlight the three aggregation mechanisms.

For the first instants of simulation, mechanism (i) predominates due to the large amount of isolated NPs: the number of clusters in the simulation box decreases sharply while their medium size remains small (see Figure 4). After the first μs, the few remaining isolated NPs collide with already formed clusters, which therefore grow (mechanism (ii)). Finally, after a few μs, there are no more isolated NPs, so only mechanism (iii) is possible and the aggregation process is slower (see Figure 4). The aggregation process is characterized by the increase of the clusters dimension, from a few up to thousands of NPs, depending on the volume fraction considered and the particle size.

The process described can be clearly observed in Figure 5, which shows the aggregation kinetic with R=0.78 nm and ϕ=3.5%: the clusters size distribution at 0.2 μs, 5 μs and 30 μs was investigated via histograms and 3D snapshots. At the beginning of the simulation, given the high mobility of dispersed NPs and small clusters, aggregation proceeds rapidly: at 0.2 μs, most NPs were grouped into small clusters, each containing 150 or fewer NPs (see Figure 5A). As the simulation proceeds, small clusters merge and reach up to 1500 NPs per aggregate, as it can see observed by comparing Figure 5A to Figure 5B,C, where the number of small aggregates progressively decreases and the probability distribution peak shifts towards larger aggregate size. The aggregation kinetics of the other configurations tested proceeded similarly, being faster or slower depending on the particle radius *R* and volume fraction ϕ.

## 4. Discussion

**Figure 6:**
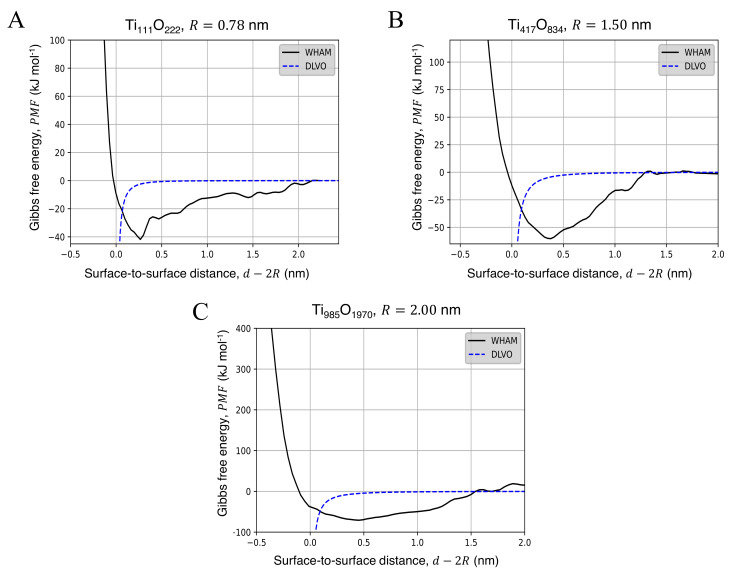
Comparison between the classic DLVO theory (dashed blue line), evaluated through Equation (7), and the PMF evaluated obtained by WHAM procedure (solid black line) for a pair of (**A**) Ti_111_O_222_ NPs, (**B**) a pair of Ti_417_O_834_ NPs and (**C**) a pair of Ti_985_O_1970_ NPs.

The fundamental theoretical background describing the interaction of surfaces immersed in a liquid the DLVO (Derjaguin-Landau-Verwey-Overbeek) theory [56,57,58], which evaluates the pair-wise potential as the combination of attractive van der Waals forces with repulsive electric forces given by the electric double layer formed at the solid-liquid interface. All the configurations considered in this work are charge-neutral, as the TiO_2_ NPs were built according to their stoichiometric composition. Therefore, we expect that most of the contribution to the stability of the suspension is given by the van der Waals attraction between particles, whereas the repulsion given by the electric double layer is negligible: the DLVO theory, in this case, predicts an unstable suspension which flocculates, just as we observed in our BD simulations. However, the well-established theories used to predict the van der Waals attraction fail to effectively describe NPs potential curves. In fact, the attraction potential between two identical spheres with radius *R* can be evaluated as [59]:

(7) $$W=-\frac{A_{H}}{6}\left[\frac{2R^{2}}{d^{2}-4R^{2}}+\frac{2R^{2}}{d^{2}}+\ln\left(\frac{d^{2}-4R^{2}}{d^{2}}\right)\right]$$

where $A_{H}$ = 6 × $10^{-20}$ J is the Hamaker constant for TiO_2_-TiO_2_ in water [59] and *d* is the distance between the centres of the spheres. The interaction potentials obtained from Equation (7) and those obtained by the MD simulations are compared in Figure 6. The DLVO potential given by Equation (7) predicts an attraction well, which asymptotically tents to −∞ as the surface-to-surface distance between the NPs tends to zero, i.e., d→2R (see blue dashed lines in Figure 6). The results of the MD simulations show that the free energy minimum appears at a larger distance with respect to d=2R: as commented above, this is the effect of the van der Waals radii of the surface atoms, which exert a strong repulsive force opposing to further approach of the NPs, and can not be modeled by Equation (7). Steric repulsion avoids the mutual penetration of NPs as they enter the region of the potential well: a direct implementation of the obtained DLVO potential would not allow the BD simulations to converge to an equilibrium configuration. These results are in line with those previously reported in the literature [50] and reaffirm the inadequacy of DLVO theory in describing the interaction potential between NPs.

The pioneering work of Smoluchowski [60] describes the time evolution of the aggregate size distribution by means of an integrodifferential equation, and sets the basis for the theoretical modeling in several processes. Considering the cluster formation (NP-NP interaction) and growth (NP-cluster and cluster-cluster interaction), the particles sticking probability, thus their interaction potential, leads to two limiting regimes: diffusion- and reaction-limited. The former considers the motion of particles and aggregates to be the main constraint to aggregation, thus considering extremely probable the aggregation of particles as soon as they come into contact, while the latter considers the opposite case. The interaction potentials obtained from the MD simulations show no energy barrier preventing particles from entering the potential well region, suggesting a diffusion-limited process. Furthermore, the results of the cluster analysis presented in Section 2.4 showed very fast aggregation kinetics at the beginning of the simulation (see Figure 4), suggesting a high probability that the particles remain attached once their collision occurs. Therefore, the comparison between the BD simulations and theoretical results was performed considering a diffusion-limited aggregation. According to Smoluchowski’s model, the average number of NPs per aggregate at time *t* can be evaluated as [61,62]:

(8) $$N_{t}=1+t/t_{p}\;,$$

where $t_{p}$ is the aggregation time constant and depends on the particles size, concentration and interaction potential, and the properties of the solvent. For a diffusion limited cluster-cluster aggregation, Einstein’s diffusion equation yields and $t_{p}$ can be expressed as [62]:

(9) $$t_{p}=\frac{\pi \mu R^{3}W}{k_{B}T\phi }\;,$$

where μ is the dynamic viscosity of the solvent and *W* is the stability ratio of the colloidal suspension. Considering the limiting case of a stable and well-dispersed colloidal suspension, the combination of strong repulsive forces (namely, a high energy barrier in the pair-wise interaction potential) and hydrodynamic NP-NP interactions limit the diffusion of the particles, leading to W≫1 and $t_{p}\to \infty$. On the opposite case, considering nano-sized particles, a negligible energy barrier and hydrodynamic interaction W→1 and $t_{p}\ll 1$. Being the simulated NP neutral and in the absence of electrolytes in the considered solution, the Stability ratio can be evaluated as:

(10) $$W=2R\int_{0}^{\infty }\frac{B(h)}{{\left(h+2R\right)}^{2}}\exp\left(\frac{W_{R}+W_{A}}{k_{B}T}\right)dh\;,$$

where h=d−2R is the surface to surface separation between a pair of NPs, B(h) is a correction coefficient to include the hydrodynamic NP-NP interaction, and $W_{A}$ and $W_{R}$ are the attractive and repulsive components of the interaction potential, respectively, and should be evaluated according to the DLVO theory. As previously stated, the system considered is charge-neutral, thus $W_{R}\approx 0$ and $W_{A}$ was evaluated through Equation (7). The hydrodynamic coefficient is commonly evaluated as [63,64]:

(11) $$B\left(h\right)=\frac{6{\left(h/R\right)}^{2}+13\left(h/R\right)+2}{6{\left(h/R\right)}^{2}+4\left(h/R\right)}$$

Equations (8)–(11) were used to evaluate the average size of the aggregates in the colloidal suspension at time *t*, considering the NPs as initially well-dispersed. Note that the adopted theoretical model does not account for a limited number of particles in the system and predicts the same size for all the aggregates present in the solution.

**Figure 7:**
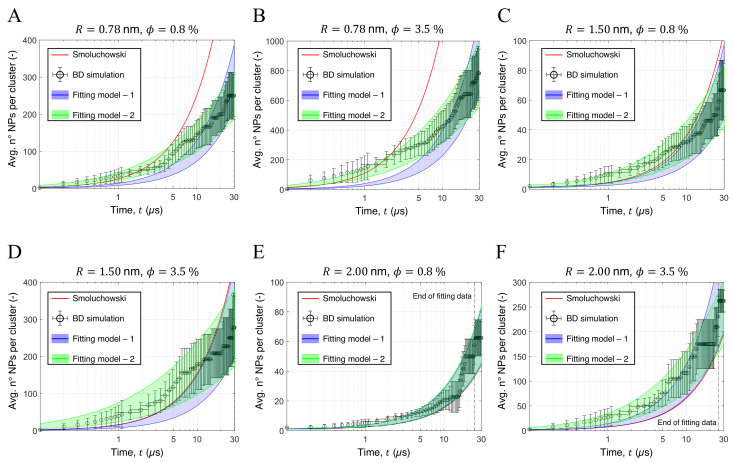
Comparison between the Smoluchowski theoretical model (solid red lines), the simulation data (black circles) and the modified aggregation models (shaded blue and green areas). The comparison was performed considering the configurations with R=0.78 nm and (**A**) ϕ=0.8% or (**B**) ϕ=3.5%; with R=1.50 nm and (**C**) ϕ=0.8% or (**D**) ϕ=3.5%; with R=2.00 nm and (**E**) ϕ=0.8% or (**F**) ϕ=3.5%. To avoid numerical artifacts in the fitting procedure, only the time frames presenting more than 5 clusters were considered by the fitting algorithm.

The theoretical predictions (solid red lines) and simulation results (black circles) of some of the tested configurations are compared in Figure 7. The setups with smaller NPs exhibit greater deviations from theoretical predictions (see Figure 7A,B for R=0.78 nm and Figure 7C,D for R=1.5 nm): while aggregation proceeds faster at low simulation times, after about 2 μs it will proceed more slowly than the Smolochowski model. This result can be interpreted considering that aggregation in highly concentrated solutions could initially proceed faster due to multi-particle collisions, whereas the Smoluchowski theory only considers binary collisions [65], and slow down at higher times due to the reduced mobility of larger aggregates. On the other hand, the largest particles tested presented faster aggregation kinetics with respect to the theoretical model (see Figure 7E,F). The discrepancy between the analytical and numerical results was evaluated by the root mean square error (RMSE), which is shown as a function of ϕ and *R* in Supplementary Figure S3. In general, the results show higher values of the RMSE for smaller NPs and larger ϕ, which is in agreement with previous results: it was shown that the classic aggregation theory works well for extremely dilute solutions, namely ϕ<0.1%, which are controlled by binary collisions, whereas are dominating in systems with larger ϕ, thus leading to faster aggregation is higher than the one predicted by the original model [65]. Clearly, when small NPs are considered, the theoretical model overestimates the mobility of large aggregates. Furthermore, this effect is enhanced at high volume fractions, which the clusters growth rate.

Thus, to recover the simulated aggregation kinetic, the theoretical model was modified by simply including a single fitting coefficient $n_{1}$ in the definition of $t_{p}$, namely:

(12) $$t_{p}^{*,1}=n_{1}t_{p}\;,$$

where $t_{p}$ can be evaluated from Equation (9). The numerical coefficient $n_{1}$ was fitted on the simulation data by the least-squares method. The results of the fitted model are shown in Figure 7 in the form of blue shaded areas, representing the model predictions in the uncertainty range given by the simulation data. As can be observed, by adding a single numerical coefficient, the aggregation kinetics of the configurations with values R=2 nm can be recovered. However, the model does not account for the progressive increase in aggregate size, thus failing to reliably predict the behavior of the configurations with R=0.78 nm and R=1.5 nm.

Thus, to provide an accurate descriptor of the aggregation process, the original aggregation model was time-discretized to include the average size of the NPs clusters in the aggregation kinetic. Considering a time-step Δt, Equation (8) was re-written to evaluate the average numbers of NPs in each aggregate $N_{i}$ at time $t_{i}=i\Delta t$.

(13) $$N_{i}=N_{i-1}+\frac{\Delta t}{t_{p}^{*,2}}\;,$$

where $t_{p}^{*,2}$ is a modified time constant used to fit the simulation results:

(14) $$t_{p}^{*,2}=\exp\left(n_{2}{\left(\frac{N_{i-1}}{\phi }\right)}^{1/3}\right)\frac{\pi \mu R^{3}W^{*}}{k_{B}T\phi }\;,$$

where $n_{2}$ is a numerical fitting coefficient and the modified stability ratio $W^{*}$ was evaluated as $W^{*}=n_{3}W$. The proposed correction allows to include the average instantaneous size of the aggregates in the evaluation of $t_{p}^{*,2}$ and to slow down the aggregation kinetic for larger values of $N_{i}$, mimicking a reduction of the aggregates mobility as their size increases. Equations (13) and (14) were iteratively solved considering $N_{i=0}=1$ and used to fit the numerical constants $0\leq n_{2}\;,n_{3}\leq 1$ on the aggregation trends reported. Clearly, by setting $n_{2}=0$ and $n_{3}=1$ the original aggregation model is recovered. Figure 7 reports the fitting results (shaded green area), while the best-fit values of $n_{2}$ and $n_{3}$ are reported in Table 3. To grant statistical representation, the fitting algorithm considered only the time frames with more than 5 aggregates in the simulation box (dotted black lines in Figure 7). As it can be observed, the proposed adapted model is in excellent agreement with the simulation data and it is suited to describe the predicted aggregation kinetic of all the configurations tested.

## 5. Conclusions

This work aimed at presenting a method for describing TiO_2_ NP aggregation in water starting from their atomistic description. The coordinates of the atoms forming the NPs were retrieved from quantum calculations. MD simulations of pairs of NPs were performed to calculate their interaction potential (PMF) using the Umbrella Sampling technique. The results obtained were compared with the theoretical PMF predicted by the DLVO theory, finding significant discrepancies and justifying the proposed approach. The PMF was used to perform BD simulations of tens of thousands of NPs at a relatively low computational cost. Such large systems could not have been studied with regular MD simulations, so the protocol adopted was crucial for understanding the aggregation kinetic of TiO_2_ NPs. Thus, the proposed protocol bridges the gap between the quantum description of a single particle and the observation of the microscopic clustering process.

Although the method was applied to TiO_2_ NPs, its generality makes it suitable for a wide variety of other materials, including charged or coated particles. In fact, the NPs considered in this work are neutral, while they might present a net non-zero surface charge arising from the positive under-coordinated Ti${}^{4+}$ and Ti${}^{5+}$ sites as well as the negative under-coordinated oxygen sites. Therefore, the case reported could be considered as a limiting case of TiO_2_ NPs at their isoelectric point, namely the value of the solution pH at which an NP has zero net electric charge. The presence of a net surface charge results in a repulsive barrier, that increases the stability of the colloidal suspension, as evidenced by experimental [66] and theoretical works [50]. From a multi-scale perspective, future work will focus on modeling under-coordinated reactive sites and their role in determining an energy barrier in PMF. This may involve moving from diffusion-limited to reaction-limited kinetics, thus allowing to test the Smoluchowski theory on a complete DLVO system. The computational bottleneck is represented by the calculation of the PMF curve and the aggregation free energy using MD and US, which is very demanding in terms of computing resources: this is unfortunately mandatory because of the inability of the DLVO theory to correctly describe the interaction between two NPs.

The aggregation kinetics obtained via BD simulations were compared with the classic Smoluchowski theory, observing larger deviations for smaller NPs at higher volume fractions. The traditional theory was time-discretized and modified to relate the aggregation kinetic with the instantaneous average cluster size, including two numerical coefficients to be fitted on the simulation data, obtaining excellent agreement. In a future perspective, increasing the number of simulations performed in the considered range of parameters would allow regressing the fitting coefficients, providing an analytical description of the aggregation kinetics consistent with the predictions of the proposed multi-scale model, for any configuration within its range of validity.

Since the scope of the H2020 project NanoInformaTIX [7] is to predict toxicity correlating the atomic structure with the toxic endpoints, in a future work, we will do BD simulations of NPs and amino acids to better describe the biological environment in contact with potentially toxic NPs. Similar studies with nanomaterials other than titanium dioxide are also being performed and will be reported in the near future.

## Figures and Tables

**Table 1:** Selected physico-chemical properties for the three molecular models. SASA is the solvent-accessible surface area, E${}_{tot}$ is the total energy in eV, $\Delta H_{f}^{\circ }$/TiO${}_{2}$ is the standard formation enthalpy (experimental value for bulk anatase is −9.78 eV [54]), E${}_{sol}$ is the solvation energy [32] in eV (not available for the largest particle).

Property	0.78 nm	1.5 nm	2.0 nm	Bulk
TiO_2_ unit formula	111	417	985	4
SASA × $10^{7}$ (m${}^{2}$)	2.15	4.44	7.14	-
E${}_{tot}$ (eV)	−2808.98	−10,623.23	−25,498.99	−105.93
$\Delta H_{f}^{\circ }$/TiO${}_{2}$ (eV)	−8.28	−8.45	−8.86	−9.46
E${}_{sol}$ (eV)	−0.42	−0.34	-	-

**Table 2:** Aggregation free energy (AFE) and its distance from the surface $d_{AFE}-2R$ of NPs pairs in water, calculated using Classical MD and US technique; the aggregation free energy was evaluated as the depth of the potential well (see Figure 2).

NP Radius, *R* (nm)	AFE (kJ mol${}^{-1}$)	$d_{\mathrm{AFE}}-2R$ (nm)
0.78	44.18	0.23
1.50	54.35	0.35
2.00	75.56	0.46

**Table 3:** Values of the numerical coefficients $n_{1}$ and $n_{2}$ used in Equations (13) and (14), obtained by minimizing the RMSE between the modelling predictions and the simulation data. The modified theoretical model allows to obtain an analytical description of the aggregation kinetic consistent with the predictions of the multi-scale model proposed.

*R* (nm)	ϕ	$n_{2}$	$n_{3}$
0.78	0.8%	0.101	0.221
	1.8%	0.101	0.362
	3.5%	0.134	0.221
	7.0%	0.148	0.101
1.50	0.8%	0.141	0.181
	1.8%	0.134	0.161
	3.5%	0.268	0.027
	7.0%	0.027	0.094
2.00	0.8%	0.0	0.698
	1.8%	0.162	0.114
	3.5%	0.169	0.067
	7.0%	0.040	0.034

## Supplementary Materials

The following are available online at https://www.mdpi.com/article/10.3390/nano12020217/s1, Classical Molecular Dynamics: simulation settings and aggregation free energy; DFT calculations: tests on bulk; Brownian Dynamics simulations: settings and cluster analysis. Figure S1: Aggregation free energy for TiO_2_ nanoparticles in water; Figure S2: Formation of clusters during the BD simulation of Ti417O834 nanoparticles with a = 0.8% reported as an example; Figure S3: Evaluation of the root mean square error (RMSE) between the theoretical aggregation kinetic, evaluated through Equations (6)–(9), and the BD simulations; Table S1: Classical Molecular Dynamics simulation parameters; Table S2: Fitting coefficients used to describe the PMFs as split-curves; Table S3: Geometry and energy results for different computational settings used in ab initio DFT calculations; Table S4: Brownian Dynamics simulation parameters; Table S5: Brownian Dynamics: number of beads.

## Abbreviations

The following abbreviations are used in this manuscript:

AFE	Aggregation Free Energy
BD	Brownian Dynamics
DFT	Density Functional Theory
EFSA	European Food Safety Authority
MD	Molcular Dynamics
NP	Nanoparticle
PMF	Potential of Mean Force
QSAR	Quantitative Structure Activity relationship
US	Umbrella Sampling
WHAM	Weighted Histogram Analysis
