# Supplementary material for: Multi-Scale Modelling of Aggregation of TiO2 Nanoparticle Suspensions in Water
Source: Nanomaterials (Basel). 2022 Jan 10;12(2):217. doi: 10.3390/nano12020217 (PMC8778026; doi:10.3390/nano12020217)
Supplement: Supplementary file 1 [file nanomaterials-12-00217-s001.zip › nanomaterials-1479938-supplementary.pdf]

Supporting Information: A multi-scale modelling of aggregation of TiO_2 nanoparticle suspensions in water

Giulia Mancardi,[*,†] Matteo Alberghini,[†,‡] Neus Aguilera-Porta,[¶] Monica Calatayud,[¶] Pietro Asinari,[†,§] and Eliodoro Chiavazzo[†]

†*Department of Energy, Politecnico di Torino, Torino, Italy*

‡*Clean Water Center, Torino, Italy*

¶*Theoretical chemistry Lab, Sorbonne Université,Paris, France*

§*Istituto Nazionale di Ricerca Metrologica, Strada delle Cacce, 91, 10135 Torino, Italy*

E-mail: giulia.mancardi@polito.it

Phone: +39 011-090-4495

Classical Molecular Dynamics simulations

Simulation settings

Table S1: Classical Molecular Dynamics simulation parameters

DL_POLY input keyword	Value
Temperature	310 K[a]
timestep	0.0001 ps
cutoff	8 Å
ewald precision	1e-6

[a] simulations made at body temperature, Nosé Hoover thermostat used with t-coupl=0.1 ps.

Aggregation free energy

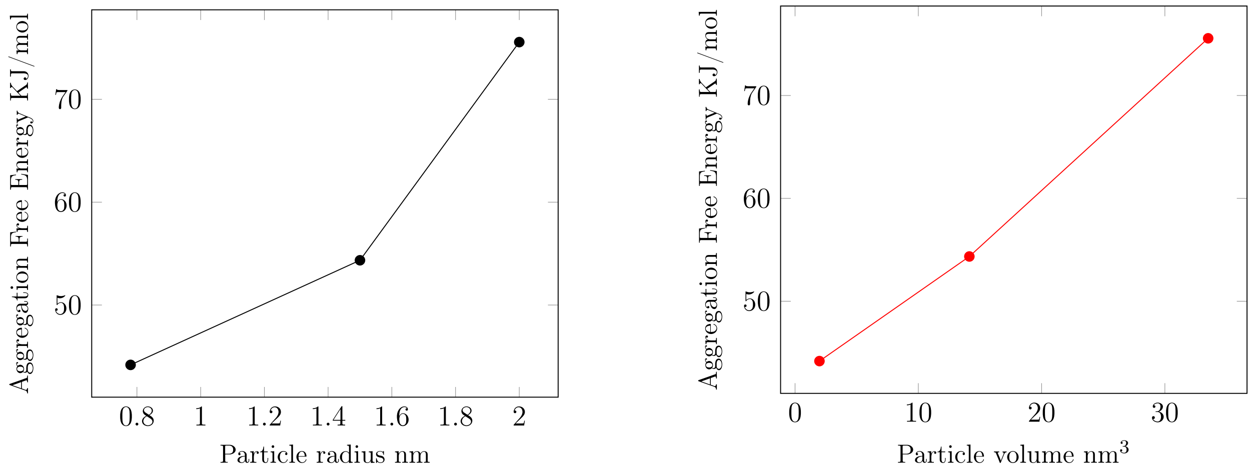

Figure S1: Aggregation free energy for TiO_2 nanoparticles in water; left panel: particle radius on x axis and free energy on y axis; right panel: particle volume on x axis and free energy on y axis.

Table S2: Fitting coefficients used to describe the PMFs as split-curves (see Fig. 2). The reported coefficients were plugged in Eq. 1 to obtain the tabled potentials used to perform the BD simulations. Note that the polynomials minima are in zero.

R (nm)	r_c (nm)	d_{AFE} (nm)	Fitting parameters				
0.78	3.50	1.79	$a_0 : +3.50e4$	$a_1 : -8.64e4$	$a_2 : +8.29e4$	$c_0 : -0.45e0$	$c_1 : 8.02e0$
			$a_3 : -3.82e4$	$a_4 : +8.25e3$	$a_5 : -6.33e2$		
			$b_0 : 0.00$	$b_1 : -5.03e1$	$b_2 : +6.26e1$		
			$b_3 : -2.67e1$	$b_4 : +4.81e0$	$b_5 : -0.31e0$		
1.5	4.20	3.35	$a_0 : +3.50e4$	$a_1 : -4.72e4$	$a_2 : +2.59e4$	$c_0 : -0.95e0$	$c_1 : 8.89e0$
			$a_3 : -7.32e3$	$a_4 : +1.07e3$	$a_5 : -6.57e1$		
			$b_0 : -8.09e4$	$b_1 : +1.05e5$	$b_2 : -5.38e4$		
			$b_3 : -1.38e4$	$b_4 : -1.75e3$	$b_5 : +8.89e1$		
2.0	5.54	4.50	$a_0 : +3.50e4$	$a_1 : +2.90e4$	$a_2 : -4.67e4$	$c_0 : -4.04e1$	$c_1 : 3.29e1$
			$a_3 : +1.88e4$	$a_4 : -3.12e3$	$a_5 : +1.90e2$		
			$b_0 : -1.82e5$	$b_1 : +1.74e5$	$b_2 : -6.65e4$		
			$b_3 : +1.26e4$	$b_4 : -1.20e3$	$b_5 : +4.53e1$		

DFT calculations

Tests on bulk

Table S3: Geometry and energy results for different computational settings used in ab initio DFT calculations. O and O_s indicate the pseudopotential. The bulk unit cell was geometrically optimized with a cutoff energy of 500 eV and 370 eV respectively, with a grid of k-points with distance 0.05 Å^{-1}. Distances in Å, energy in eV.

property	O	O_s	experimental
a	3.900	3.896	3.782
c	9.7286	9.754	9.502
$\Delta H^{\circ}{}_f$	-8.91	-9.45	-9.77[1]

Brownian Dynamics simulations

Simulation settings

Table S4: Brownian Dynamics simulation parameters

Gromacs input keyword	r=0.78 nm	r=1.5 nm	r=2 nm
ref-t	310 K[a]	310 K[a]	310 K[a]
timestep	0.1 ps		
γ: bd-fric	6096.586 amu/ps[b]	11799.844 amu/ps[b]	15733.126 amu/ps[b]
tau-t	1.454 ps[c]	2.823 ps[c]	5.000 ps[c]

[a] simulations made at body temperature;
[b] $\gamma = 6\pi\mu R_i/u$, see Eq. 3.;
[c] mass/γ.

[1] CRC Handbook of Chemistry and Physics, 88th Edition

Table S5: Brownian Dynamics: number of beads

Volume Fraction ψ	Number of particles		
	r=0.78 nm	r=1.5 nm	r=2 nm
7.0 %	36500	5000	2110
3.5 %	18000	2500	1050
1.8 %	9000	1250	550
0.8 %	4000	600	250

Cluster analysis

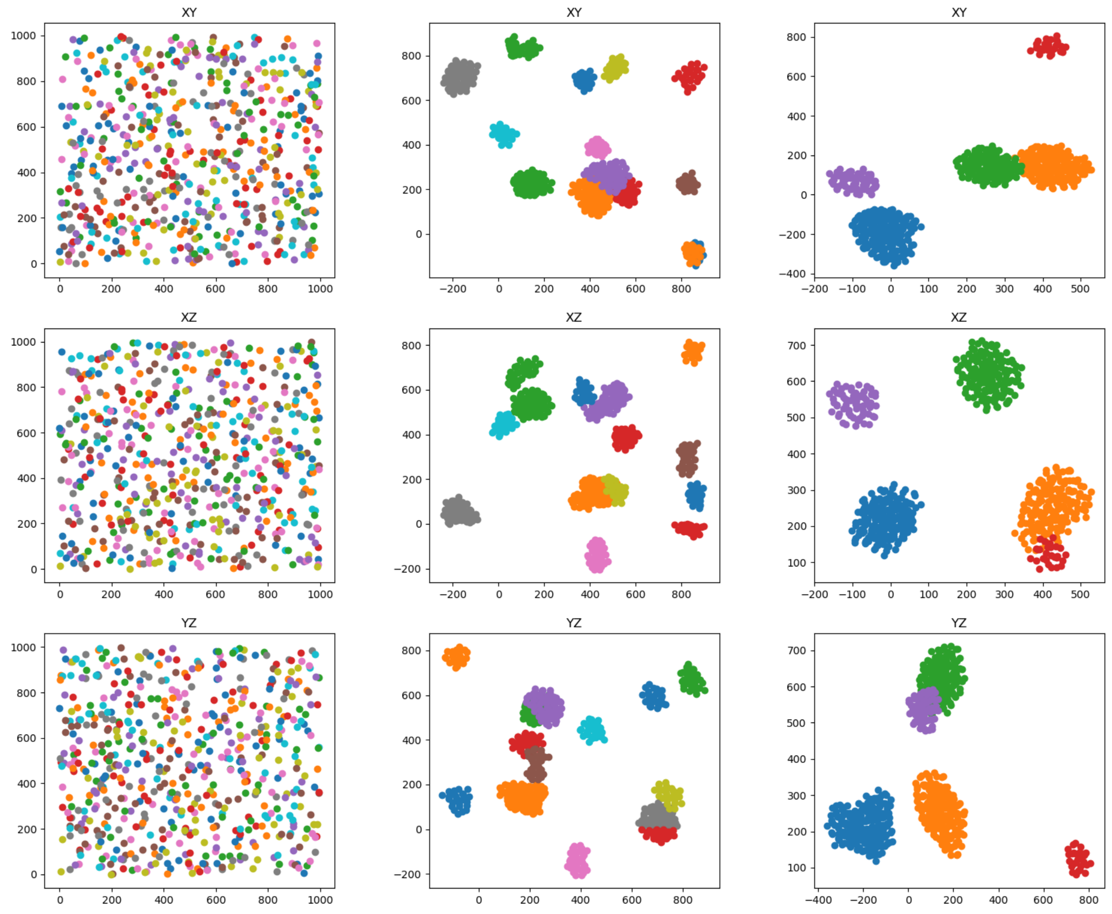

Figure S2: Formation of clusters during the BD simulation of $Ti_{417}O_{834}$ nanoparticles with a ψ=0.8% reported as an example; (a) Initial configuration with isolated NPs; (b) clusters after 0.2 μs; (c) clusters after 1 μs. 3-dimensional clusters are analyzed using DBSCAN algorithm on xy, xz and yz planes for simplicity.

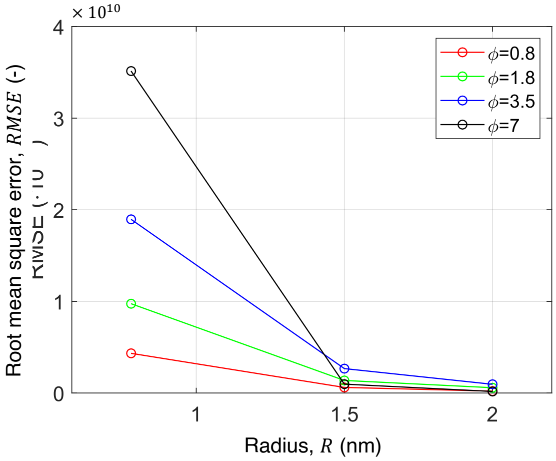

Figure S3: Evaluation of the root mean square error (RMSE) between the theoretical aggregation kinetic, evaluated through Eqs. 6-9, and the BD simulations. Larger values of the RMSE are related to larger deviations from the theoretical predictions.
